# Electrophysiologic Conservation of Epicardial Conduction Dynamics After Myocardial Infarction and Natural Heart Regeneration in Newborn Piglets

**DOI:** 10.3389/fcvm.2022.829546

**Published:** 2022-03-09

**Authors:** Hanjay Wang, Terrence Pong, Oluwatomisin O. Obafemi, Haley J. Lucian, Joy Aparicio-Valenzuela, Nicholas A. Tran, Danielle M. Mullis, Stefan Elde, Yuko Tada, Sam W. Baker, Caroline Y. Wang, Kevin J. Cyr, Michael J. Paulsen, Yuanjia Zhu, Anson M. Lee, Y. Joseph Woo

**Affiliations:** ^1^Department of Cardiothoracic Surgery, Stanford University, Stanford, CA, United States; ^2^Stanford Cardiovascular Institute, Stanford University, Stanford, CA, United States; ^3^Department of Cardiovascular Medicine, Stanford University, Stanford, CA, United States; ^4^Department of Comparative Medicine, Stanford University, Stanford, CA, United States; ^5^Department of Bioengineering, Stanford University, Stanford, CA, United States

**Keywords:** heart, regeneration, neonate - age, myocardial infarction, electrophysiology, conduction, mapping

## Abstract

Newborn mammals, including piglets, exhibit natural heart regeneration after myocardial infarction (MI) on postnatal day 1 (P1), but this ability is lost by postnatal day 7 (P7). The electrophysiologic properties of this naturally regenerated myocardium have not been examined. We hypothesized that epicardial conduction is preserved after P1 MI in piglets. Yorkshire-Landrace piglets underwent left anterior descending coronary artery ligation at age P1 (*n* = 6) or P7 (*n* = 7), After 7 weeks, cardiac magnetic resonance imaging was performed with late gadolinium enhancement for analysis of fibrosis. Epicardial conduction mapping was performed using custom 3D-printed high-resolution mapping arrays. Age- and weight-matched healthy pigs served as controls (*n* = 6). At the study endpoint, left ventricular (LV) ejection fraction was similar for controls and P1 pigs (46.4 ± 3.0% vs. 40.3 ± 4.9%, *p* = 0.132), but significantly depressed for P7 pigs (30.2 ± 6.6%, *p* < 0.001 vs. control). The percentage of LV myocardial volume consisting of fibrotic scar was 1.0 ± 0.4% in controls, 9.9 ± 4.4% in P1 pigs (*p* = 0.002 vs. control), and 17.3 ± 4.6% in P7 pigs (*p* < 0.001 vs. control, *p* = 0.007 vs. P1). Isochrone activation maps and apex activation time were similar between controls and P1 pigs (9.4 ± 1.6 vs. 7.8 ± 0.9 ms, *p* = 0.649), but significantly prolonged in P7 pigs (21.3 ± 5.1 ms, *p* < 0.001 vs. control, *p* < 0.001 vs. P1). Conduction velocity was similar between controls and P1 pigs (1.0 ± 0.2 vs. 1.1 ± 0.4 mm/ms, *p* = 0.852), but slower in P7 pigs (0.7 ± 0.2 mm/ms, *p* = 0.129 vs. control, *p* = 0.052 vs. P1). Overall, our data suggest that epicardial conduction dynamics are conserved in the setting of natural heart regeneration in piglets after P1 MI.

## Introduction

Ischemic heart disease affects 200 million people and results in 10 million deaths globally each year (1). Although revascularization techniques have improved early survival after acute myocardial infarction (MI), many survivors develop heart failure despite receiving optimal management (2). The low proliferative capacity of the adult mammalian cardiomyocyte may contribute to the heart's inability to recover after suffering massive cardiomyocyte death after MI (3). A variety of adjunctive therapies (e.g., stem cells, small molecules, angiogenic cytokines, engineered tissues, and various biomaterials) have been developed to stimulate or support cardiac repair after MI (4), although none have achieved widespread clinical translation. Stem cell therapies, in particular, may be arrhythmogenic due to imperfect electromechanical coupling of transplanted cells with the native myocardium (5), thus illustrating the importance of assessing the electrophysiologic effects of any cardioregenerative therapy.

In the healthy mammalian heart, electrical impulses are transmitted from the sinoatrial node to the atrioventricular node to the bundle of His. Electrical conduction then passes rapidly within the interventricular septum via the right and left bundle branches, emerging epicardially first as two wavefront foci on the free walls of the right and left ventricles, respectively, before radiating and converging to activate the mid-ventricle and apex via the Purkinje fiber network, and finally reaching the basal posterolateral region last (6, 7). After MI, however, the development of fibrotic scar alters impulse propagation within and around the infarct (8). Moreover, anteroseptal MI resulting from occlusion of the proximal left anterior descending (LAD) coronary artery may result in more global ventricular conduction delays and an increased QRS interval on electrocardiography, as the blood supply of the right bundle branch, the anterior fascicle of the left bundle branch, and, to a lesser extent, the posterior fascicle of the left bundle branch, may all depend on septal perforators from the LAD (9).

Natural heart regeneration was first characterized in urodele amphibians (e.g., newts) and teleost fish (e.g., zebrafish), resulting in minimal scar after ventricular apical resection (10, 11). In mammals, including mice (12–14), rats (15), rabbits (16), pigs (17, 18), and possibly humans (19), natural heart regeneration is a transient phenomenon exhibited only by newborns. Neonatal mammals which suffer MI on postnatal day 1 (P1) activate an intrinsic cardiomyocyte proliferation response that ultimately generates renewed myocardium and preserves cardiac function (20). By postnatal day 7 (P7), however, mammals appear to lose this ability, and the injured myocardium is replaced with fibrotic scar, resulting in ventricular dysfunction, similar to that observed in adult mammals.

Although natural heart regeneration represents a promising therapeutic strategy for the treatment of ischemic heart disease, the electrophysiologic properties of this regenerated myocardium have not been examined in mammals. Recent advances in flexible electronics have created the opportunity to perform high-resolution electrophysiologic mapping of the epicardial surface (21, 22). These tools allow for detailed spatiotemporal characterization of electrical activity on the surface of the heart, and facilitate comparison of electrophysiologic properties between normal and diseased cardiac models *in vivo* (22). Here, we performed epicardial mapping on piglet hearts *in vivo* and hypothesized that epicardial conduction is preserved after MI in P1 piglets in the setting of natural heart regeneration.

## Methods

### Experimental Design

Neonatal piglets underwent LAD ligation at age P1 (*n* = 11) or age P7 (*n* = 14) to induce MI. A subset of P1 (*n* = 3) and P7 (*n* = 5) piglets were sacrificed immediately after LAD ligation to assess area at risk. All other P1 (*n* = 8) and P7 piglets (*n* = 9) were recovered. At the designated study endpoint 7 weeks after surgery, a total of 6 P1 pigs (75.0% survival, including 3 males, 3 females) and 7 P7 pigs (77.8% survival, including 3 males, 4 females) were alive, at which point cardiac magnetic resonance imaging (MRI) was performed, followed by epicardial conduction mapping and heart explant for histopathologic analysis. Epicardial mapping data was available for all 6 P1 pigs and for 5 P7 pigs (including 2 males, 3 females). Healthy 7–8-week-old weight-matched pigs (*n* = 6, including 2 males, 4 females) served as controls for cardiac MRI, epicardial conduction mapping, and histopathology.

### Animal Care and Use

Pregnant Yorkshire-Landrace sows (Pork Power Farms, Turlock, CA, USA) were housed individually and monitored for parturition every 12 h. Piglets were cared for by their nursing sow until weaned at 3 weeks old and subsequently housed in groups of 2-3 animals without separation by sex. Healthy 7–8-week-old Yorkshire-Landrace pigs were separately obtained (Pork Power Farms) and housed similarly as the weaned pigs. Food and water were provided *ad libitum*. All experiments were performed in accordance with the National Institutes of Health Guide for the Care and Use of Laboratory Animals 8th Edition and approved by the Institutional Animal Care and Use Committee at Stanford University (Protocol 33527).

### Neonatal Piglet MI Model

**Figure 1:**
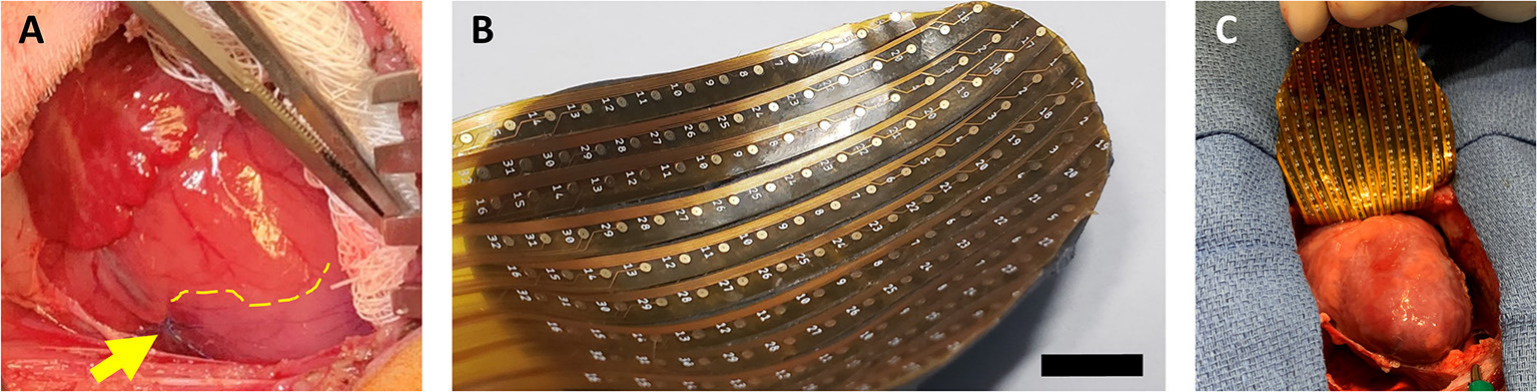
Neonatal piglet myocardial infarction model and epicardial mapping. **(A)** Permanent ligation of the left anterior descending coronary artery (yellow arrow) was performed in piglets on postnatal day 1 or postnatal day 7 to induce myocardial infarction. The ischemic territory (below dotted yellow line) was visibly distinguished from the perfused myocardium by pallor and hypokinesis. **(B,C)** After 7 weeks, epicardial conduction mapping was performed using custom 3D-printed high-resolution mapping arrays with contoured shells matching the epicardial surface profile of the 7–8-week-old pig heart, ensuring optimal coverage and contact during mapping. Scale bar, 1 cm.

LAD ligation was performed by one surgeon. Intramuscular ketamine (5 mg/kg) was administered for anesthetic induction, followed by intramuscular buprenorphine (0.01 mg/kg). The piglets were then endotracheally intubated, and general anesthesia was maintained using inhaled 3–5% isoflurane. Intravenous cefazolin (25 mg/kg) was given for antibiotic prophylaxis. A left anterolateral thoracotomy was performed, and a 6-0 polypropylene suture was placed around the LAD immediately distal to the take-off of the first diagonal branch. The suture was crossed to assess the territory at risk. The LAD was then permanently ligated, and infarction of the anterior wall and apex was confirmed by pallor and hypokinesis (Figure 1A), in association with new ST-segment elevation changes. The chest was closed in layers. Subcutaneous bupivacaine liposome injectable suspension (5.3 mg/kg) was administered for perioperative analgesia. The piglets were recovered, and iron dextran (100 mg) was administered intramuscularly. After all piglets in the litter had recovered, they were returned to the care of the sow. Intramuscular carprofen (3 mg/kg) was additionally administered daily for 2 days after surgery.

### Assessment of Area at Risk

Immediately after LAD ligation, a subset of piglets was allocated for assessment of area at risk. A median sternotomy was performed, and the heart was arrested by intravenous delivery of potassium chloride (1 mEq/kg). The ascending aorta was clamped, and 50 mL of 2% Evans blue solution (Cat: E2129, Sigma-Aldrich, St. Louis, MO, USA) was delivered into the coronary arteries via the aortic root. The heart was then explanted and serially washed in normal saline. The left ventricle (LV) was isolated and sliced transversely into short-axis sections with 5 mm thickness from the apex to the base. As previously described (23), the blue-stained region of each slice represents the perfused myocardium, while the unstained region of each slice represents the area at risk. The unstained region of each slice was cut away using a scalpel and the total weight of the unstained regions for each heart was recorded. The total weight of the blue-stained regions for each heart was also recorded. The area at risk for each heart was calculated as a percentage of the total LV as follows:


$$\begin{array}{l} \%\;Area\;at\;Risk\\ =\;\frac{\sum Weight\;of\;Unstained\;Regions}{\sum Weight\;of\;Blue\;Regions+\;\sum Weight\;of\;Unstained\;Regions} \end{array}$$

### Cardiac MRI

At 7 weeks after surgery, the pigs were placed under general anesthesia via intramuscular tiletamine-zolazepam (6 mg/kg) and buprenorphine (0.005 mg/kg), and endotracheally intubated. Cardiac MRI was performed using a Signa HDx 3.0 T MRI scanner (GE Healthcare, Chicago, IL, USA) and an 8-channel chest coil. Cine and late gadolinium enhancement (LGE) imaging were acquired under electrocardiographic gating and breath-holding. Cine images were obtained with the fast imaging employing steady-state acquisition sequence and array sensitive encoding technique (repetition time 3.4 ms; echo time min-full; flip angle 45°; thickness 6 mm; matrix 224 x 224; field of view 26 cm). Next, 15 min after administration of intravenous gadobenate dimeglumine (0.2 mmol/kg, Multihance, Bracco Diagnostics Inc., Monroe Township, NJ, USA), LGE images were obtained with the fast gradient echo-inversion recovery sequence (repetition time 6.2 ms; echo time 2.9 ms; flip angle 15°; thickness 6 mm; matrix 224 x 192; field of view 26 cm; inversion time 280–350 ms). MRI analysis was performed using Medis Suite 3.0 (Medis Medical Imaging Systems Inc., Raleigh, NC, USA). Endocardial and epicardial contours were traced semi-automatically to quantify LV end-diastolic volume (EDV), end-systolic volume (ESV), ejection fraction (EF), stroke volume (SV), cardiac output, and muscular volume. LGE representing fibrotic scar after MI was identified in three-dimensional (3D) space as signals exceeding 5 standard deviations from that of healthy remote myocardium (QMass 8.1, Medis Medical Imaging Systems Inc.).

### Epicardial Conduction Mapping and Terminal Surgery

Following cardiac MRI, the pigs were brought to the operating room. A median sternotomy was performed, and the epicardial surface of the ventricles was fully exposed. Minimal pericardial adhesions were encountered. Epicardial conduction mapping was performed using custom 3D-printed high-resolution mapping arrays. Using cardiac MRI data, contoured shells matching the ventricular epicardial surface profile of the 7–8-week-old pig heart were generated in computer-aided design software (Fusion 360, AutoDesk, San Rafael, CA, USA) and printed with a stereolithographic printer using flexible photopolymer resin (Form3, Formlabs, Somerville, MA, USA). The shell was then fitted with high-resolution gold-plated electrode arrays (Figure 1B). With the heart in sinus rhythm (90–120 beats per min), epicardial mapping was performed for a continuous period of 60 s (Figure 1C). Following data acquisition, the heart was arrested by intravenous delivery of potassium chloride (1 mEq/kg). Finally, the heart was explanted and weighed.

### Assessment of LV Endocardium

The LV of each explanted heart was opened along the posterior septum. Digital photographs of the LV endocardium were obtained and infarct size as a percentage of total LV endocardial area was measured using digital planimetry in ImageJ (version 1.53, National Institutes of Health, Bethesda, MD, USA), as previously described (24, 25).

### Masson's Trichrome Staining

Transverse strips of anterior LV tissue at the mid-papillary level were excised, frozen in optimum cutting temperature compound (Fisher HealthCare, Cat: 23730571, Houston, TX, USA) using 2-methyl butane on dry ice, and stored at −80 °C. In a subset of pigs from the control (*n* = 4), P1 (*n* = 4), and P7 groups (*n* = 5), short-axis sections (10 μm thickness) of the anterior LV were stained with Masson's trichrome (American MasterTech, Cat: KTMTR2PT, Lodi, CA, USA), as previously described (15, 26). Images were acquired using an EVOS XL Core Imaging System (Thermo Fisher Scientific, Cat: AMEX-1000, Waltham, MA, USA). Areas of fibrosis and areas of healthy myocardium were quantified using ImageJ.

### Analysis of Epicardial Mapping Data

Electrogram data was recorded using a 256-channel biopotential measurement system (2,048 Hz sample rate, BioSemi, Amsterdam, Netherlands) and exported to MATLAB (Mathworks, Natick, MA, USA) for data analysis. Unipolar electrograms were bandpass filtered (1–400 Hz) and activation times were determined by identifying maximum absolute voltage and maximum dV/dt_max_. Channels with poor contact or noise were removed and the data interpolated. Isochrone activation maps depict activation times of the entire mapped ventricular epicardium, with red indicating early activation and blue indicating late activation. Conduction velocity was calculated using the triangulation technique (27). QRS interval was determined as the average QRS length across all channels over a representative period of 10 consecutive cycles.

### Statistical Analysis

Statistical analyses were performed using Stata version 14.2 (StataCorp LLC., College Station, TX, USA). Data are reported in the order of control vs. P1 vs. P7, unless otherwise specified. Continuous data are presented as mean ± standard deviation and compared using the two-sample *t*-test or one-way analysis of variance with Tukey's multiple comparison test for pairwise comparisons. *P* < 0.05 was considered statistically significant.

## Results

### P1 Piglets Naturally Recover LV Function After MI

**Figure 2:**
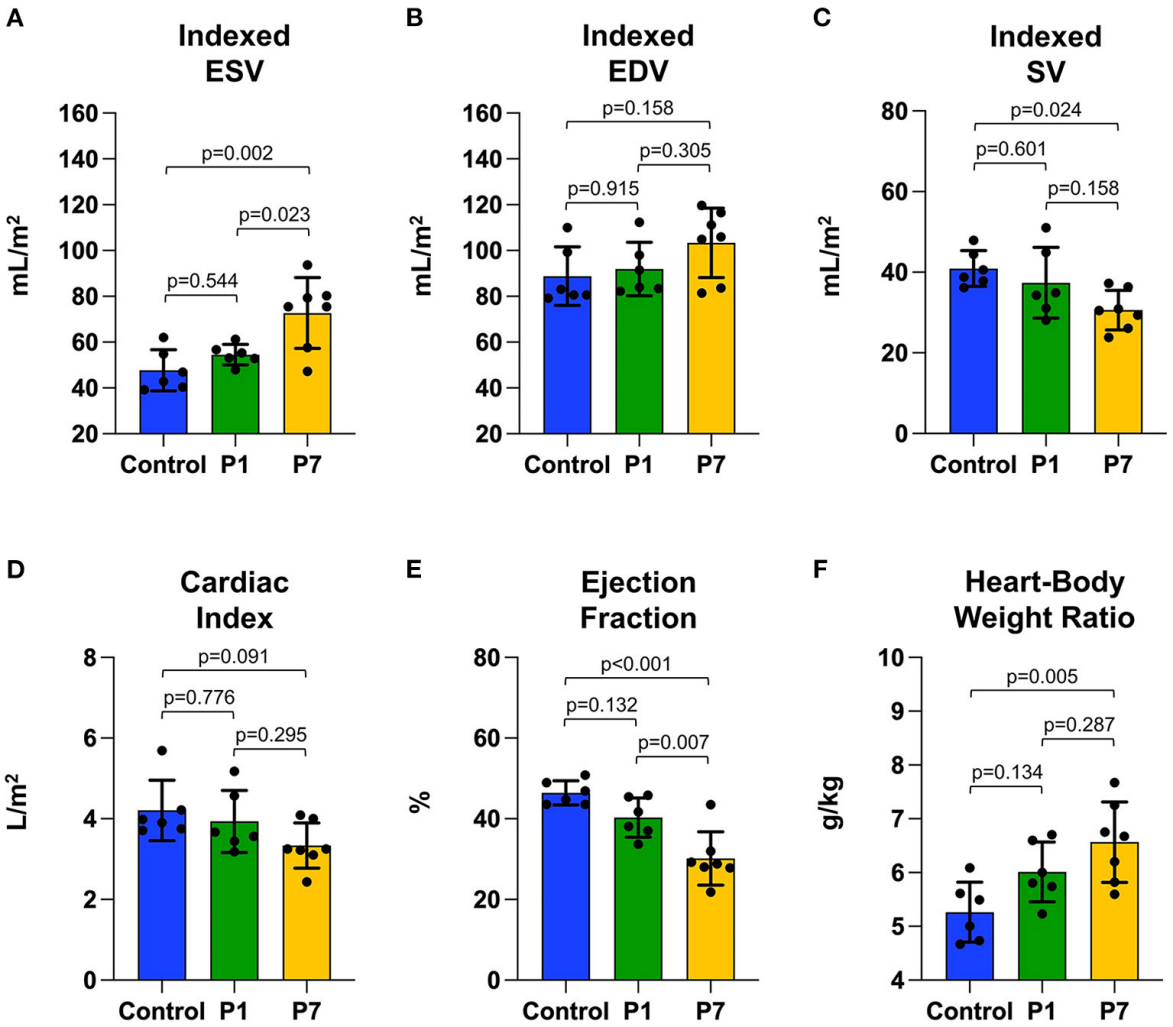
Piglets naturally recover left ventricular function after myocardial infarction on postnatal day 1. At 7 weeks after myocardial infarction (MI), cardiac magnetic resonance imaging was performed to evaluate left ventricular function, including **(A)** end-systolic volume (ESV) indexed against body surface area, **(B)** end-diastolic volume (EDV) indexed against body surface area, **(C)** stroke volume (SV) indexed against body surface area, **(D)** cardiac index, and **(E)** ejection fraction. Following heart explant, the **(F)** heart weight to body weight ratio was determined. No significant differences were observed between pigs receiving MI on postnatal day 1 (P1) compared to healthy controls. Pigs receiving MI on postnatal day 7 (P7), however, developed left ventricular dysfunction.

At the study endpoint, control and P1 pigs exhibited similar weight (12.0 ± 0.9 vs. 11.9 ± 1.7 kg, *p* = 0.989) while P7 pigs tended to have lower weight (10.5 ± 1.5 kg, *p* = 0.151 vs. control). There was no difference in heart rate between the groups (102.5 ± 9.8 vs. 107.0 ± 16.1 vs. 108.9 ± 5.6 beats per min, *p* = 0.585). After normalizing for body surface area (28), cardiac MRI revealed that control and P1 pigs had similar indexed LV ESV (47.7 ± 9.0 vs. 54.5 ± 4.5 mL/m^2^, *p* = 0.544, Figure 2A), similar indexed LV EDV (88.8 ± 12.8 vs. 91.9 ± 11.7 mL/m^2^, *p* = 0.915, Figure 2B), similar indexed SV (40.9 ± 4.4 vs. 37.4 ± 8.8 mL/m^2^, *p* = 0.601, Figure 2C), and similar cardiac index (4.2 ± 0.8 vs. 3.9 ± 0.8 L/min/m^2^, *p* = 0.776, Figure 2D). In contrast, P7 pigs exhibited an increased indexed LV ESV (72.7 ± 15.4 mL/m^2^, *p* = 0.002 vs. control), increased indexed LV EDV (103.3 ± 15.2 mL/m^2^, *p* = 0.158 vs. control), decreased indexed SV (30.6 ± 4.9 mL/m^2^, *p* = 0.024 vs. control), and decreased cardiac index (3.3 ± 0.6 L/min/m^2^, *p* = 0.091 vs. control). Although LV EF for P1 pigs was mildly decreased compared to controls (46.4 ± 3.0 vs. 40.3±4.9%, *p* = 0.132, Figure 2E), P7 pigs exhibited significantly depressed LV EF (30.2 ± 6.6%, *p* < 0.001 vs. control, *p* = 0.007 vs. P1). Similarly, P1 pigs had a mildly increased heart-body weight ratio compared to controls (5.3 ± 0.6 vs. 6.0 ± 0.6 g/kg, *p* = 0.134, Figure 2F), while P7 pigs exhibited a significantly increased heart-body weight ratio (6.6 ± 0.8 g/kg, *p* = 0.005 vs. control).

### P1 Piglets Exhibit Reduced LV Scar Formation After MI

**Figure 3:**
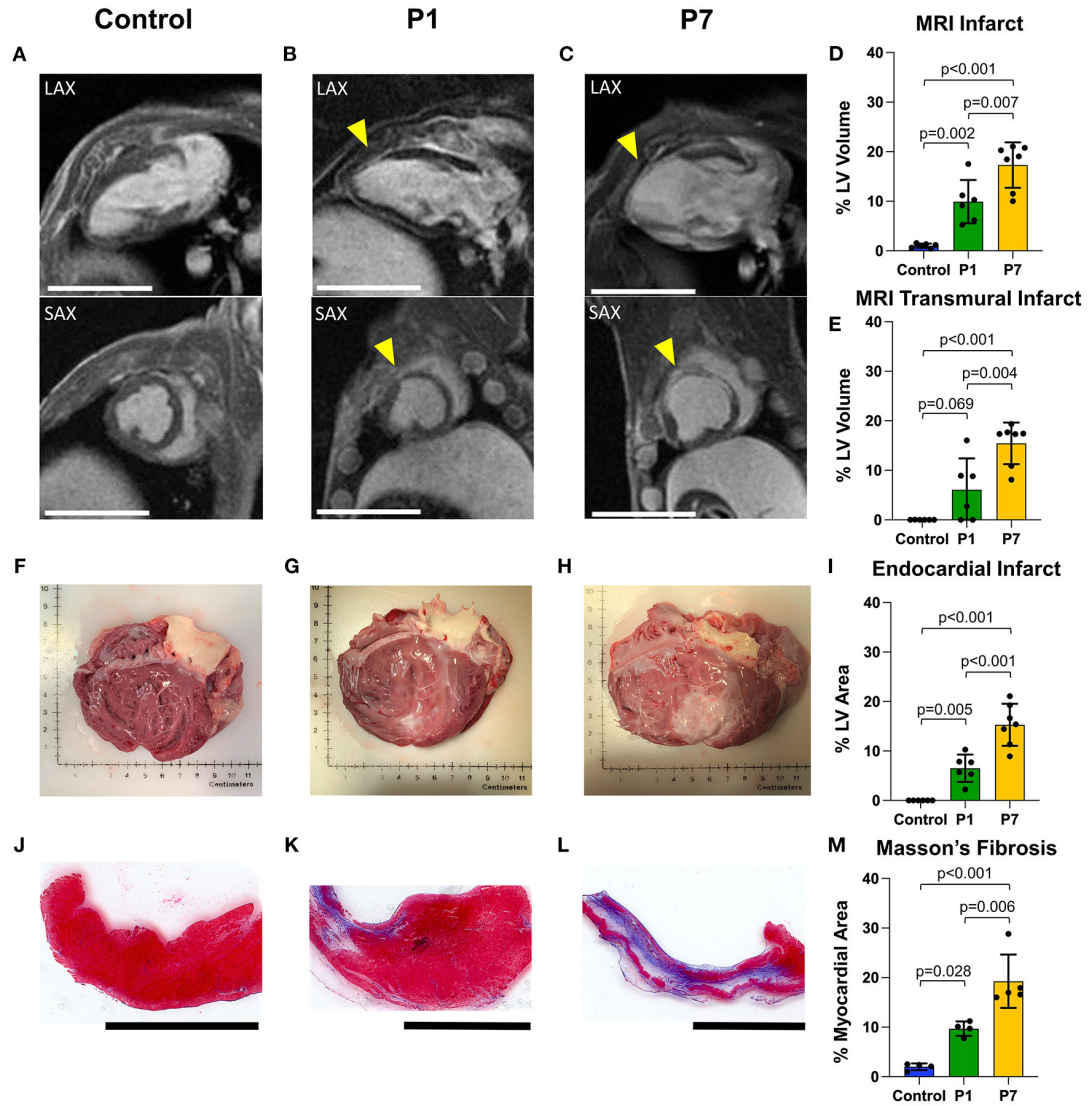
Piglets exhibit reduced scar formation after myocardial infarction on postnatal day 1. At 7 weeks after myocardial infarction (MI), cardiac magnetic resonance imaging (MRI) with late gadolinium enhancement was performed to evaluate left ventricular (LV) fibrosis. Representative long-axis (LAX) and short-axis (SAX) views are presented for **(A)** healthy controls, **(B)** pigs receiving MI on postnatal day 1 (P1), and **(C)** pigs receiving MI on postnatal day 7 (P7). Infarcted areas are marked by yellow arrows. Scale bars, 5 cm. **(D)** Compared to P7 pigs, P1 pigs developed significantly smaller infarcts, including **(E)** significantly smaller transmural infarcts. **(F–H)** Following heart explant, the LV endocardium was assessed for visible scar formation, revealing **(I)** significantly smaller endocardial scar in P1 pigs compared to P7 pigs. **(J–L)** Masson's trichome staining was performed to confirm the presence of fibrotic scar. Scale bars, 1 cm. **(M)** Significantly less fibrotic content was observed in P1 pigs compared to P7 pigs.

Immediately after LAD ligation, there was no significant difference in the area at risk for P1 vs. P7 piglets (31.4 ± 7.3% vs. 28.7 ± 8.6%, *p* = 0.666, Supplementary Figure 1). At 7 weeks after MI, infarct size was assessed using cardiac MRI with LGE, with long-axis and short-axis LV views revealing healthy myocardium in controls (Figure 3A), a small degree of anteroseptal scar but preserved LV geometry in P1 pigs (Figure 3B), and extensive anteroseptal scar with LV dilation in P7 pigs (Figure 3C). The percentage of LV myocardial volume consisting of fibrotic scar was 1.0 ± 0.4% in controls, 9.9 ± 4.4% in P1 pigs (*p* = 0.002 vs. control), and 17.3 ± 4.6% in P7 pigs (*p* < 0.001 vs. control, *p* = 0.007 vs. P1, Figure 3D), and the percentage of transmural scar was 0.0 ± 0.0% in controls, 6.1 ± 6.3% in P1 pigs (*p* = 0.069 vs. control), and 15.4 ± 4.2% in P7 pigs (*p* < 0.001 vs. control, *p* = 0.004 vs. P1, Figure 3E). Examination of explanted hearts confirmed no endocardial LV scar in controls (Figure 3F), small infarcts with preserved LV geometry in P1 pigs (Figure 3G), and large infarcts with LV dilation in P7 pigs (Figure 3H). The percentage of LV endocardial area comprised of scar was 0.0 ± 0.0% in controls, 6.5 ± 2.8% in P1 pigs (*p* = 0.005 vs. control), and 15.3 ± 4.2% in P7 pigs (*p* < 0.001 vs. control, *p* < 0.001 vs. P1, Figure 3I). Finally, the collagen composition of the LV scar was confirmed using Masson's trichome staining (Figures 3J–L), with the percentage of fibrotic area within each histologic section being 2.0 ± 0.7% for controls, 9.7 ± 1.4% for P1 pigs (*p* = 0.028 vs. control), and 19.3 ± 5.4% for P7 pigs (*p* < 0.001 vs. control, *p* = 0.006 vs. P1, Figure 3M).

### P1 Piglets Demonstrate Conserved Epicardial Conduction Dynamics After MI

**Figure 4:**
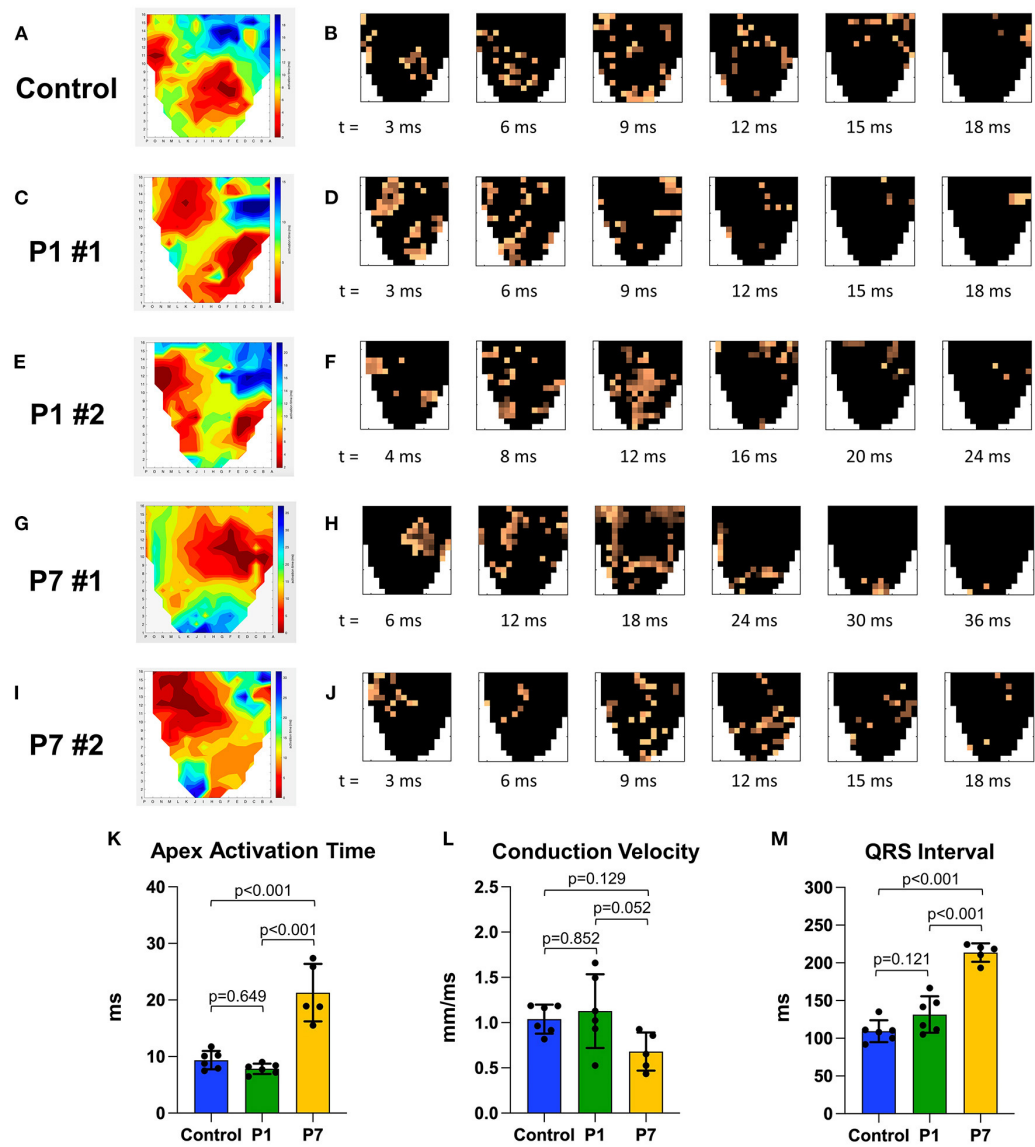
Piglets demonstrate conserved epicardial conduction dynamics after myocardial infarction on postnatal day 1. At 7 weeks after myocardial infarction (MI), epicardial conduction mapping was performed. Representative isochrone activation maps and timelapse activation maps are presented for **(A,B)** one healthy control, illustrating normal conduction through the right and left bundle branches and Purkinje system; **(C–F)** two pigs receiving MI on postnatal day 1 (P1) with similar activation patterns as healthy controls; and **(G–J)** two pigs receiving MI on postnatal day 7 (P7), including **(G,H)** one P7 pig which developed right bundle branch block, and **(I,J)** one P7 pig which developed left bundle branch block. **(K)** Apex activation time was similar between controls and P1 pigs, but profoundly delayed in P7 pigs. **(L)** Conduction velocity was similar between controls and P1 pigs, but slower in P7 pigs. **(M)** QRS interval was similar between controls and P1 pigs, but significantly longer in P7 pigs.

Isochrone and timelapse activation maps are shown for a representative healthy control in Figures 4A,B, illustrating conduction through the right and left bundle branches and normal propagation from the mid-ventricle and apex toward the basal posterolateral region. Representative maps for two P1 pigs are shown in Figures 4C–F, revealing a similar epicardial activation pattern as healthy controls. In contrast, all P7 MI isochrones exhibited left or right bundle branch blocks. Representative maps are shown for two P7 pigs, including one with right bundle branch block (Figures 4G,H) and another with left bundle branch block (Figures 4I,J), both with delayed activation of the infarcted apex. Apex activation time was similar between control and P1 pigs (9.4 ± 1.6 vs. 7.8 ± 0.9 ms, *p* = 0.649), but was significantly prolonged in P7 pigs (21.3 ± 5.1 ms, *p* < 0.001 vs. control, *p* < 0.001 vs. P1, Figure 4K). The conduction velocity was also similar between control and P1 pigs (1.0 ± 0.2 vs. 1.1 ± 0.4 mm/ms, *p* = 0.852), but slower in P7 pigs (0.7 ± 0.2 mm/ms, *p* = 0.129 vs. control, *p* = 0.052 vs. P1, Figure 4L). Finally, whereas the QRS length for control pigs was 109.4 ± 14.5 ms, the QRS length for P1 pigs was mildly increased at 131.5 ± 24.0 ms (*p* = 0.121 vs. control), and the QRS length for P7 pigs was significantly prolonged at 213.5 ± 12.3 ms (*p* < 0.001 vs. control, *p* < 0.001 vs. P1, Figure 4M). Across all animals, a direct relationship was observed for QRS length as a linear function of LV fibrosis (R^2^ = 0.548, Supplementary Figure 2).

## Discussion

In this study, we performed the first electrophysiologic assessment of naturally regenerated myocardium in a neonatal mammalian MI model. Our data confirmed that P1 pigs recover LV function and exhibit limited scar formation after LAD ligation. Furthermore, epicardial activation pattern, apex activation time, conduction velocity, and QRS interval remained similar between P1 pigs and healthy controls. In contrast, P7 pigs developed LV dysfunction and formed large scars after LAD ligation, in association with aberrant epicardial activation, prolonged apex activation time, slower conduction velocity, and lengthened QRS interval. These results suggest that natural heart regeneration is able to conserve native epicardial conduction dynamics after MI.

The bioelectric environment of the injured heart recovering after MI has been a subject of longstanding research interest. While the infarct zone was historically believed to be electrically inert, it is now known that remnants of surviving myocardium persist within the infarct zone (29), and that fibroblasts comprising the scar can electrically couple with cardiomyocytes (30), altogether allowing scar tissue to retain some residual conductive capacity. However, conduction via myocyte-fibroblast coupling is limited in range to only ~300 μm (31), and the scattered areas of remnant myocardium within the infarct zone are not structurally organized, leading to slow conduction velocity and nonuniform propagation around the scar (8, 29). Strategies to improve conduction dynamics within the injured myocardium may include enhancing the ability of fibroblasts to electrically couple with cardiomyocytes, for example by overexpressing gap junction proteins such as connexin43 (32), or by increasing the ratio of surviving cardiomyocytes to fibroblasts in the infarct zone, for example by stimulating cardiomyocyte proliferation or by reducing infarct size.

Natural heart regeneration repopulates the infarcted region with new cardiomyocytes and results in significantly reduced infarct size (20), thereby providing a viable substrate for maintaining normal electrical conduction after myocardial injury. A previous study using optical voltage mapping after ventricular apical resection in zebrafish demonstrated significantly delayed conduction near the apex at 1 week after injury, followed by gradual recovery of normal isochrone density and conduction velocity by 4 weeks after injury, suggesting full electrical coupling between native and regenerated cardiomyocytes (33). Using a more clinically relevant LAD ligation model to induce ischemic injury instead of traumatic injury, as well as a more translationally relevant porcine model to examine natural heart regeneration in a large mammal with similar cardiac anatomy and physiology as humans, we likewise observed that pigs exhibited similar isochrone activation maps and conduction velocities as healthy controls at 7 weeks after P1 MI. This normalization of electrical conduction after P1 MI in piglets was achieved despite the presence of a small residual scar, the size of which was similar to that of a previous report (17). It is possible that, akin to how cardiac biomechanical properties may be preserved by myocardial regeneration despite the presence of a small infarct remaining after MI (26, 34, 35), there may also be a threshold of scar formation below which ventricular conduction dynamics are not significantly disrupted.

While cardiomyocytes, fibroblasts, endothelial cells, and immune cells have all been studied in the context of natural heart regeneration (36–38), little is known about how the cells of the cardiac conduction system respond during natural heart regeneration. Studies in adult dogs and pigs have shown that Purkinje fiber cells are more resistant to ischemic injury than cardiomyocytes (39, 40). Nevertheless, in adult pigs, Purkinje cell populations sharply decline after acute MI, and the number of Purkinje-myocardial junctions remains chronically decreased at 1 month after MI, even if early reperfusion is achieved (40). Interestingly, a recent report by Kahr et al. described an expansion of Purkinje cell density at 10 days and at 4 weeks after LAD ligation in neonatal mice (41). Kahr et al. additionally showed that the Purkinje cells did not exhibit significantly increased cell cycle activity in response to ischemia, but that preexisting cardiomyocytes may be recruited to become new Purkinje cells during the process of natural heart regeneration. RNA sequencing of the neonatal mouse Purkinje cells after MI further revealed strong upregulation of genes involved in inflammation, chemotaxis, and angiogenesis, including *Endothelin1*, which is known to govern the trans-differentiation of cardiomyocytes into Purkinje cells during embryonic cardiac development (42). These data suggest that, during natural heart regeneration after MI in neonatal mammals, preexisting cardiomyocytes are not only the source of new cardiomyocytes to maintain normal myocardial architecture and ventricular function (20), but potentially also the source of new conducting cells in the His-Purkinje system to maintain normal conduction dynamics as well.

Some cell therapies have been shown to significantly reduce scar size after MI, but their potential arrhythmogenicity remains a clinical concern (43). The causes of arrhythmias after cell therapy may include imperfect electrical coupling between transplanted cells and native cardiomyocytes (e.g., insufficient gap junction connectivity), poor excitability of transplanted cells (e.g., low density of requisite ion channels for depolarization), intrinsic electrophysiologic differences between transplanted cells and native cardiomyocytes (e.g., different action potential phase lengths), heightened automaticity of transplanted cells (e.g., override of the sinoatrial node pacemaker), and effects related to cell delivery techniques (e.g., inflammation and fibrosis due to mechanical trauma of intramyocardial injection) (5). Because naturally regenerated cardiomyocytes are derived from preexisting native cardiomyocytes, it is possible that their innate genetic and phenotypic similarities allow them to more homogeneously integrate into existing conduction pathways compared to relatively foreign transplanted progenitors. As transcriptomic analyses begin to unveil the mechanisms underlying neonatal cardiac regeneration (44, 45), future strategies to activate endogenous regenerative pathways may arise through targeted genetic or molecular therapies. It will be important to investigate each of the above concerns with respect to the relationship between native and naturally regenerated cardiac cells, including the quantity and quality of gap junctions, and the compatibility of their electrophysiologic profiles for automaticity, excitation, and propagation.

Several limitations of our study must be noted. First, as prior work had already established the regenerative capacity of the newborn piglet heart (17, 18), and traced the origin of new Purkinje cells after MI in neonatal mice to preexisting cardiomyocytes (41), our study focused on assessing epicardial conduction dynamics in neonatal piglets after MI and was not designed to investigate the mechanisms underlying natural heart regeneration and its effects on the conduction system. It is likely that cardiomyocyte regeneration, reduced scar size, and cardiomyocyte-Purkinje cell trans-differentiation all contribute to preserving conduction after MI in newborn mammals, and future work will endeavor to determine the relative contribution of each. In addition, our study involved small sample sizes, which limited our ability to resolve potential differences between the experimental groups. Indeed, although we did not observe any statistically significant differences in LV function or QRS length between the control and P1 groups, we nevertheless acknowledge that some differences may become apparent with larger sample sizes, possibly due to residual scarring after P1 MI. Another limitation of our study was the single timepoint for analysis. Although it would be interesting to explore the evolution of electrical conduction dynamics, including the development of arrhythmias, over multiple timepoints in individual animals, conduction mapping experiments require terminal surgery, and disturbing the piglet nursery prior to weaning age caused significant stress to the sow and risked injury to the piglets. Finally, it is important to note that we used healthy age- and weight-matched pigs as our control group instead of performing sham surgery, as sham littermates may outcompete the MI piglets during the critical weeks of growth before weaning. As a result, we are not able to account for any potential effects of postoperative inflammation or mechanical trauma associated with needle passage through the myocardium during LAD ligation on scar formation and epicardial conduction.

Overall, our data suggest that epicardial conduction dynamics are conserved in the setting of natural heart regeneration in piglets after P1 MI. These findings support the need for continued investigation of therapeutic modalities that target natural regenerative pathways. While this study focused on the electrophysiologic profile of naturally regenerated myocardium at the tissue level, future experiments will characterize the cellular electrophysiology of naturally regenerated cardiomyocytes, confirm electromechanical coupling between preexisting and regenerated cardiomyocytes, and further explore the putative mechanisms of scar size reduction vs. cardiomyocyte-His/Purkinje cell trans-differentiation in preserving electrical conduction dynamics.

## Data Availability Statement

The raw data supporting the conclusions of this article will be made available by the authors, without undue reservation.

## Ethics Statement

The animal study was reviewed and approved by Institutional Animal Care and Use Committee, Stanford University.

## Author Contributions

HW and YW conceived the study. HW, TP, OO, DM, MP, YZ, AL, and YW designed the experiments. HW, TP, OO, HL, JA-V, NT, DM, YT, SB, and KC performed the experiments. HW, TP, OO, HL, JA-V, NT, DM, SE, YT, and CW analyzed the data. AL and YW provided experimental resources. HW, OO, AL, and YW provided funding for the study. HW wrote the first draft of the manuscript. All authors contributed significantly to the revision of the manuscript. All authors read and approved the submitted manuscript.

## Funding

This work was funded in part by the National Institutes of Health (R01HL089315-11 to YW; R38HL14361501 to OO), the American Heart Association (18POST33990223 to HW), and by a Stanford Bio-X Interdisciplinary Initiatives Seed Grant and Stanford Cardiovascular Institute Seed Grant. Animal experiments were performed in Stanford University shared facilities supported by NIH S10RR029020-01. Finally, we would like to thank Mr. M. Ian Ritchie for the generous donation to support this study.

## Conflict of Interest

The authors declare that the research was conducted in the absence of any commercial or financial relationships that could be construed as a potential conflict of interest.

## Publisher's Note

All claims expressed in this article are solely those of the authors and do not necessarily represent those of their affiliated organizations, or those of the publisher, the editors and the reviewers. Any product that may be evaluated in this article, or claim that may be made by its manufacturer, is not guaranteed or endorsed by the publisher.

## Supplementary Material

Supplementary Figure 1 Area at risk after left anterior descending coronary artery ligation in neonatal piglets. Immediately following ligation of the left anterior descending coronary artery, the myocardial area at risk (AAR) was calculated as a percentage of the entire left ventricle (LV) by weight using Evans blue staining, revealing no significant difference in AAR after surgery on postnatal day 1 (P1) vs. postnatal day 7 (P7).

Supplementary Figure 2 Correlation between QRS length and degree of left ventricular fibrosis after myocardial infarction in neonatal piglets. A scatterplot of QRS length vs. degree of left ventricular (LV) fibrosis is shown, including healthy controls (blue) and piglets at 7 weeks after myocardial infarction on postnatal day 1 (P1, green) and postnatal day 7 (P7, yellow). A direct relationship is observed between QRS length as a linear function of LV fibrosis.
