# Supplementary figures and images for: Electrophysiologic Conservation of Epicardial Conduction Dynamics After Myocardial Infarction and Natural Heart Regeneration in Newborn Piglets
Source: Front Cardiovasc Med. 2022 Mar 9;9:829546. doi: 10.3389/fcvm.2022.829546 (PMC8959497; doi:10.3389/fcvm.2022.829546)

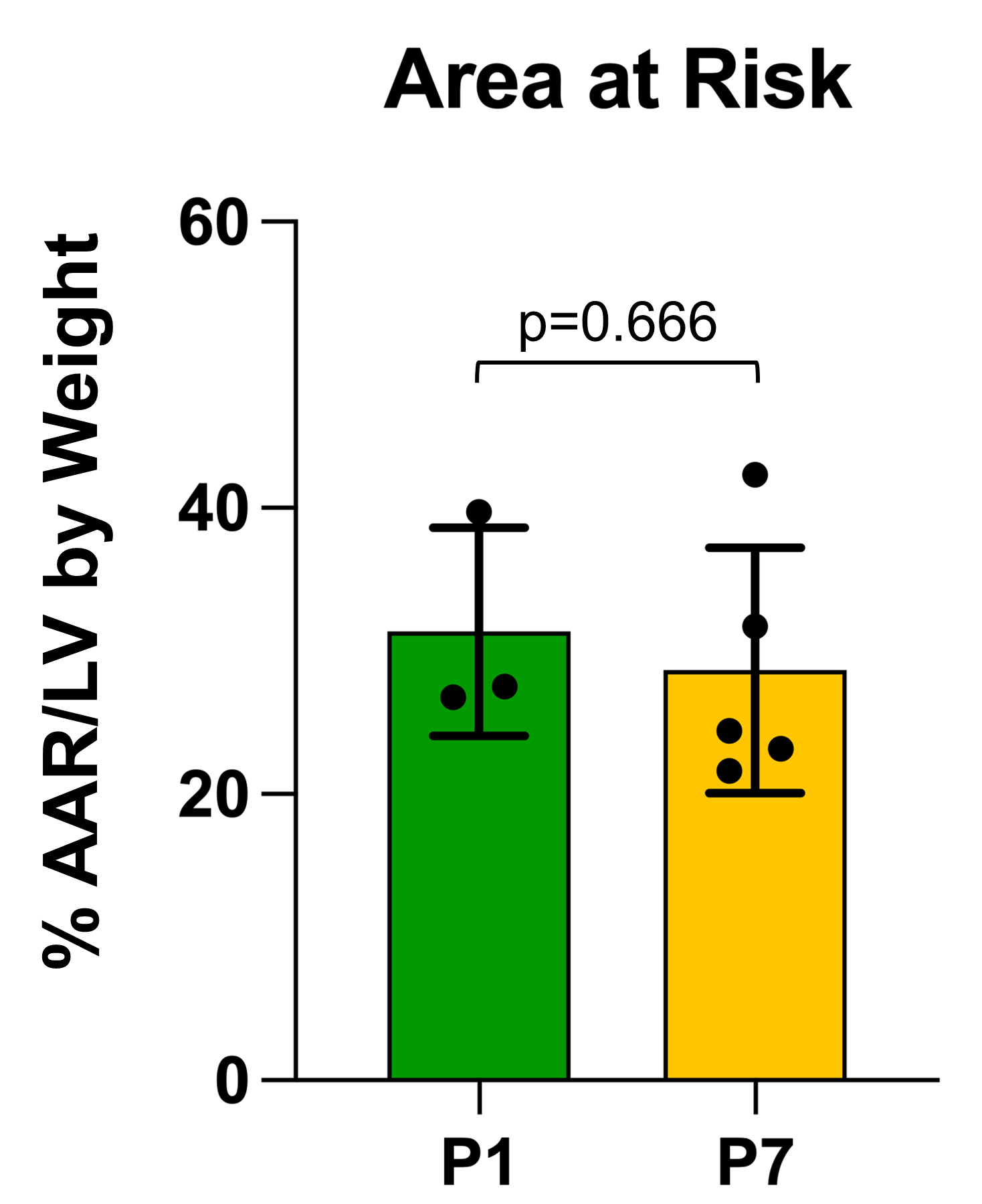

Supplement: Supplementary Figure 1 — Area at risk after left anterior descending coronary artery ligation in neonatal piglets. Immediately following ligation of the left anterior descending coronary artery, the myocardial area at risk (AAR) was calculated as a percentage of the entire left ventricle (LV) by weight using Evans blue staining, revealing no significant difference in AAR after surgery on postnatal day 1 (P1) vs. postnatal day 7 (P7). [file Image_1.TIF]

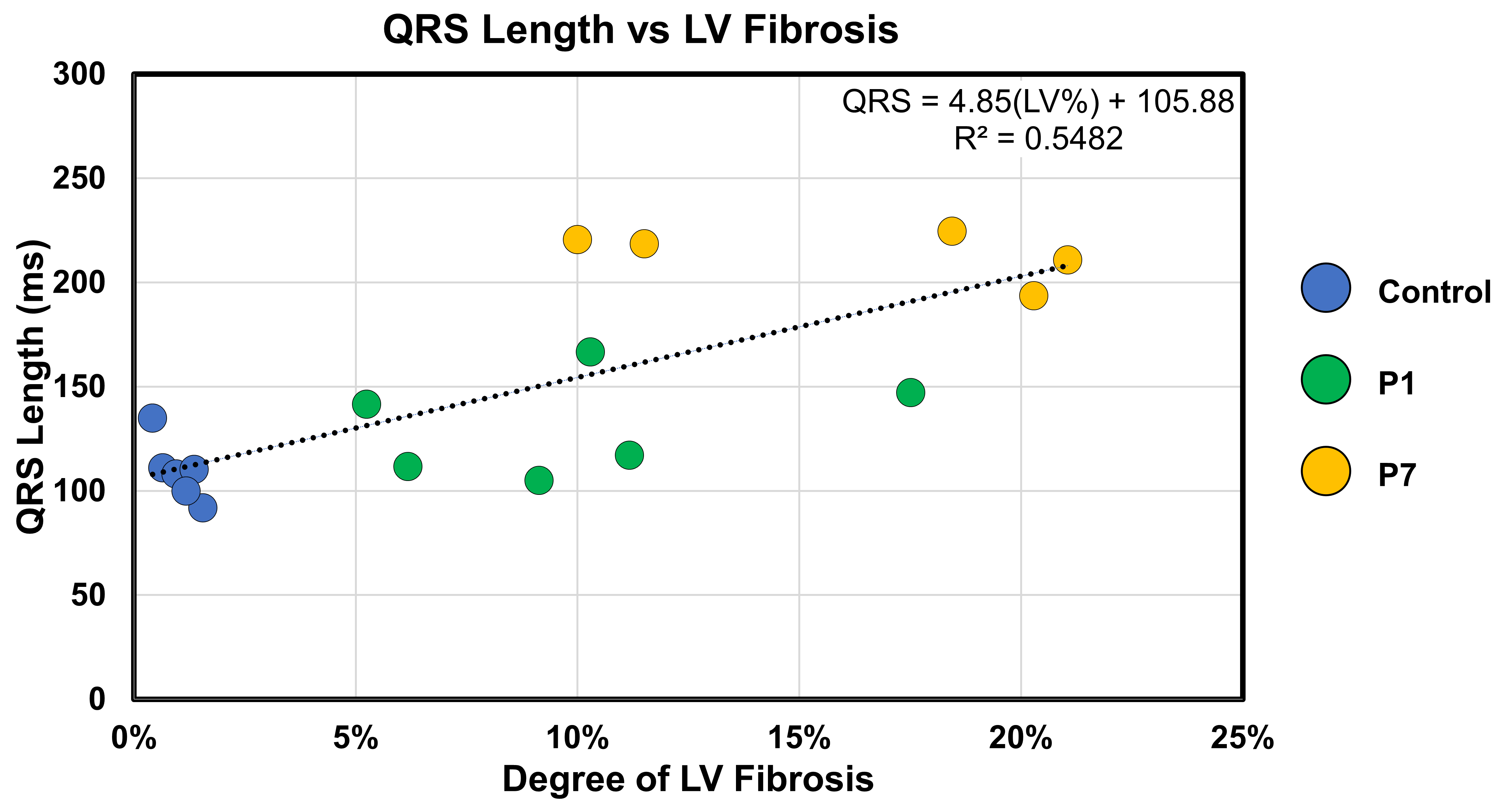

Supplement: Supplementary Figure 2 — Correlation between QRS length and degree of left ventricular fibrosis after myocardial infarction in neonatal piglets. A scatterplot of QRS length vs. degree of left ventricular (LV) fibrosis is shown, including healthy controls (blue) and piglets at 7 weeks after myocardial infarction on postnatal day 1 (P1, green) and postnatal day 7 (P7, yellow). A direct relationship is observed between QRS length as a linear function of LV fibrosis. [file Image_2.TIF]
